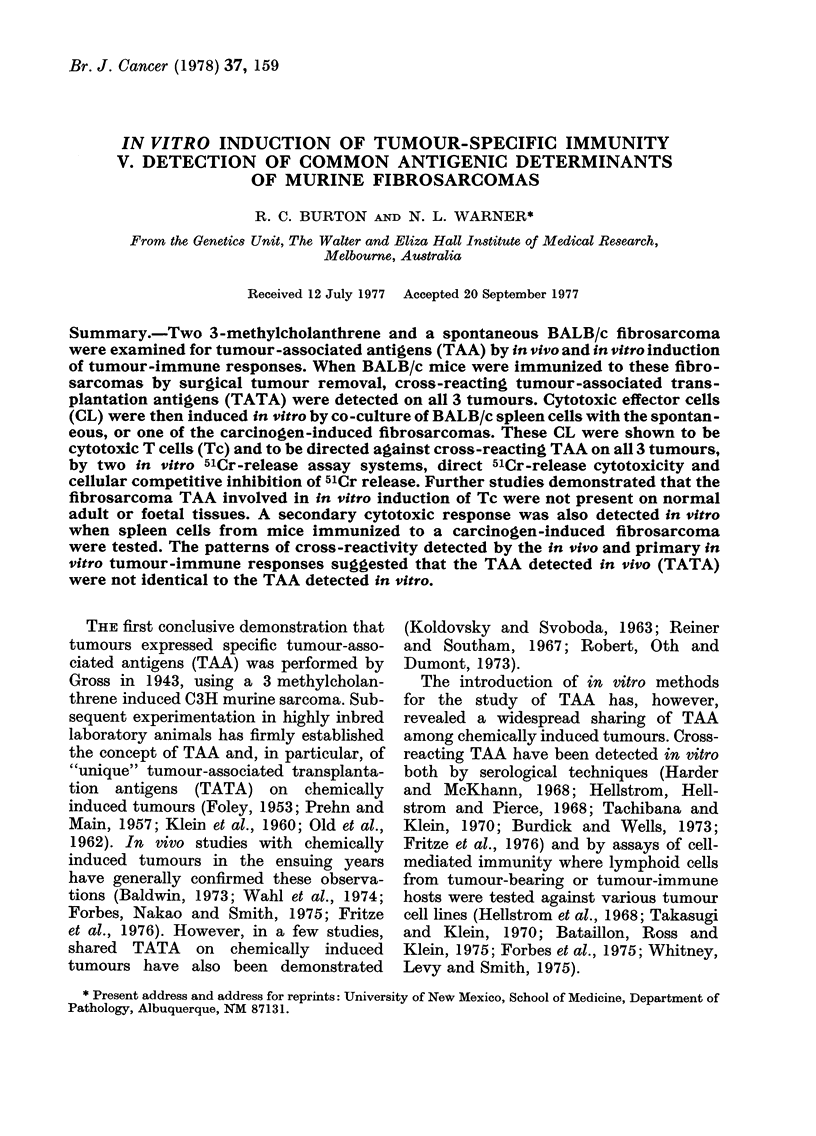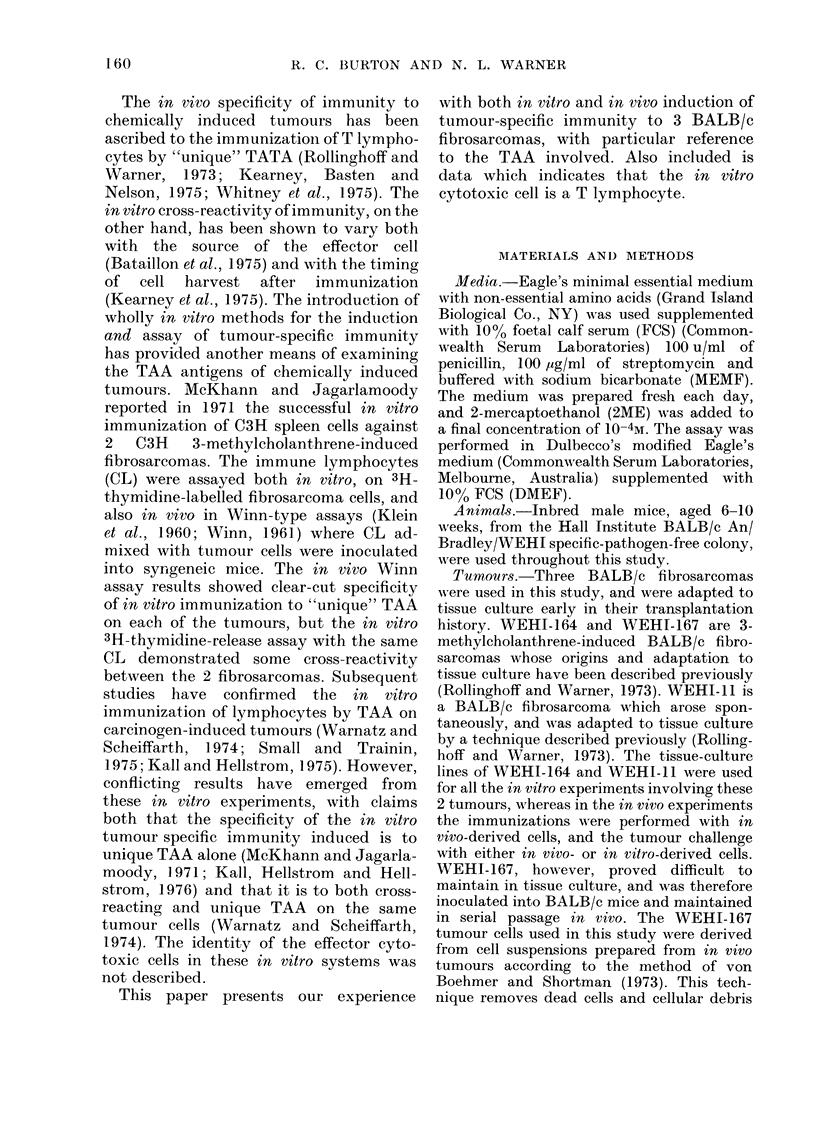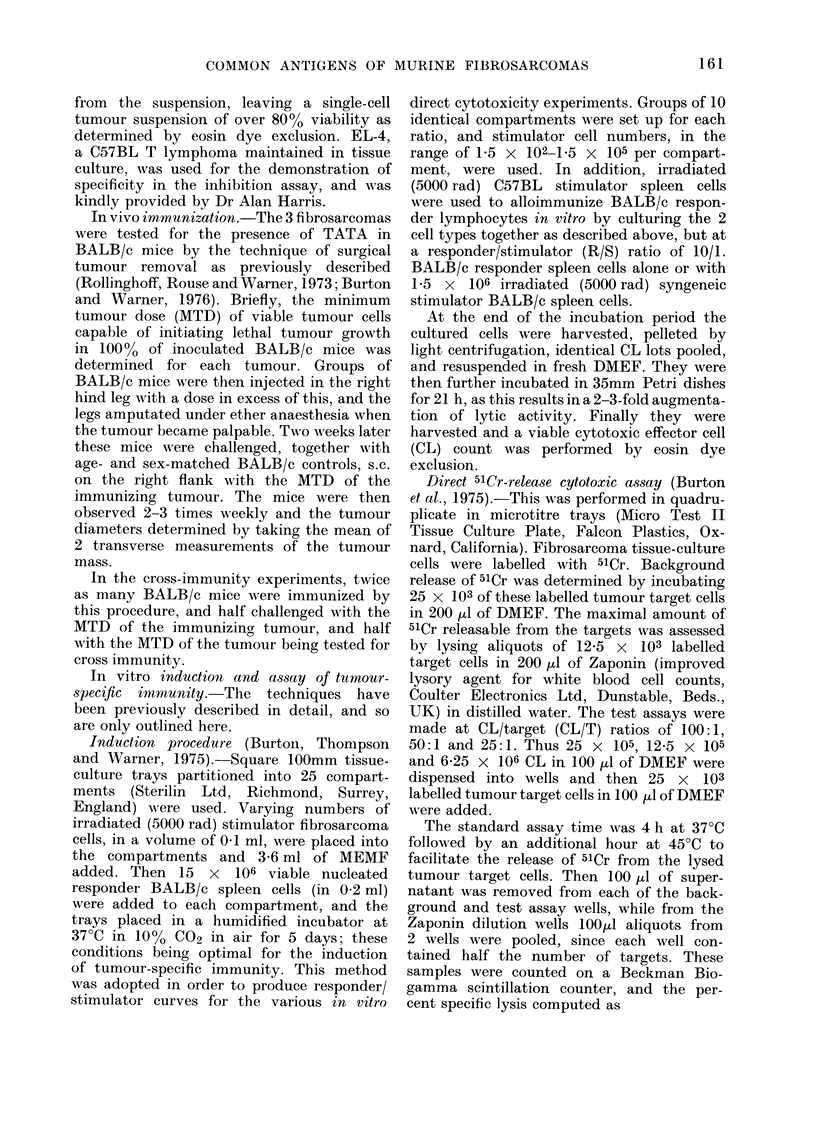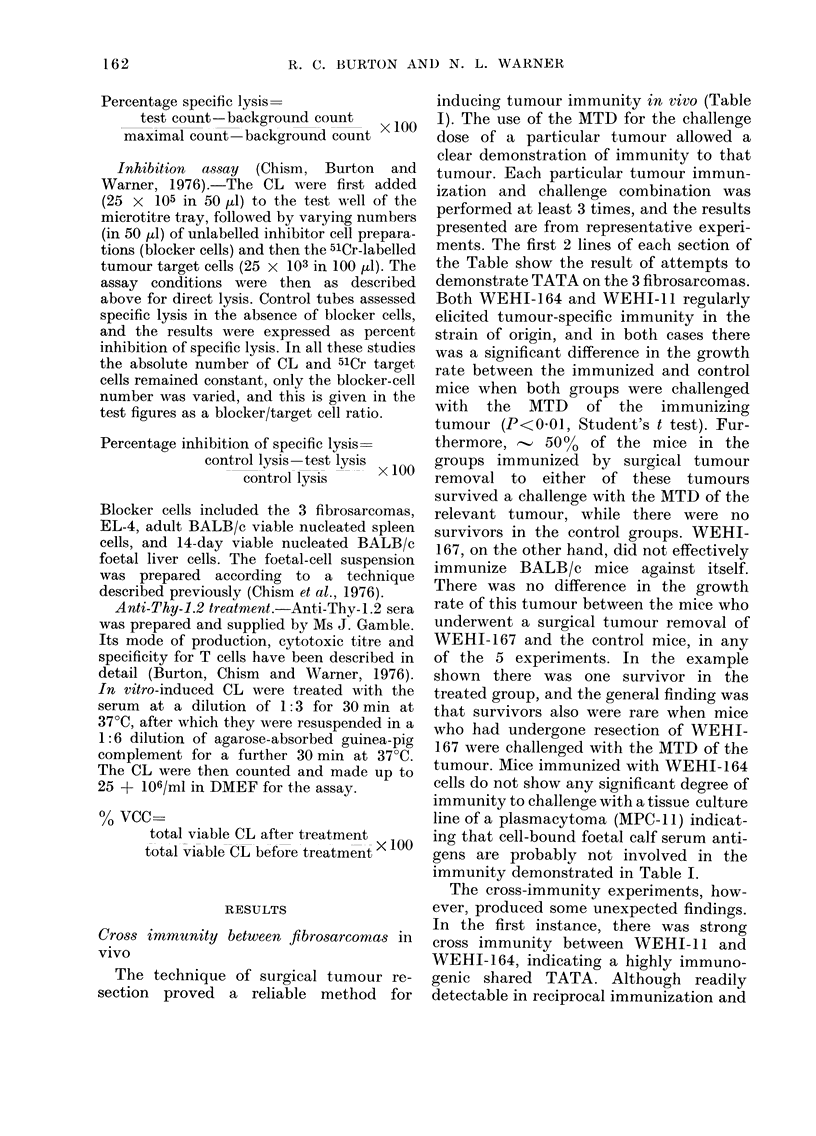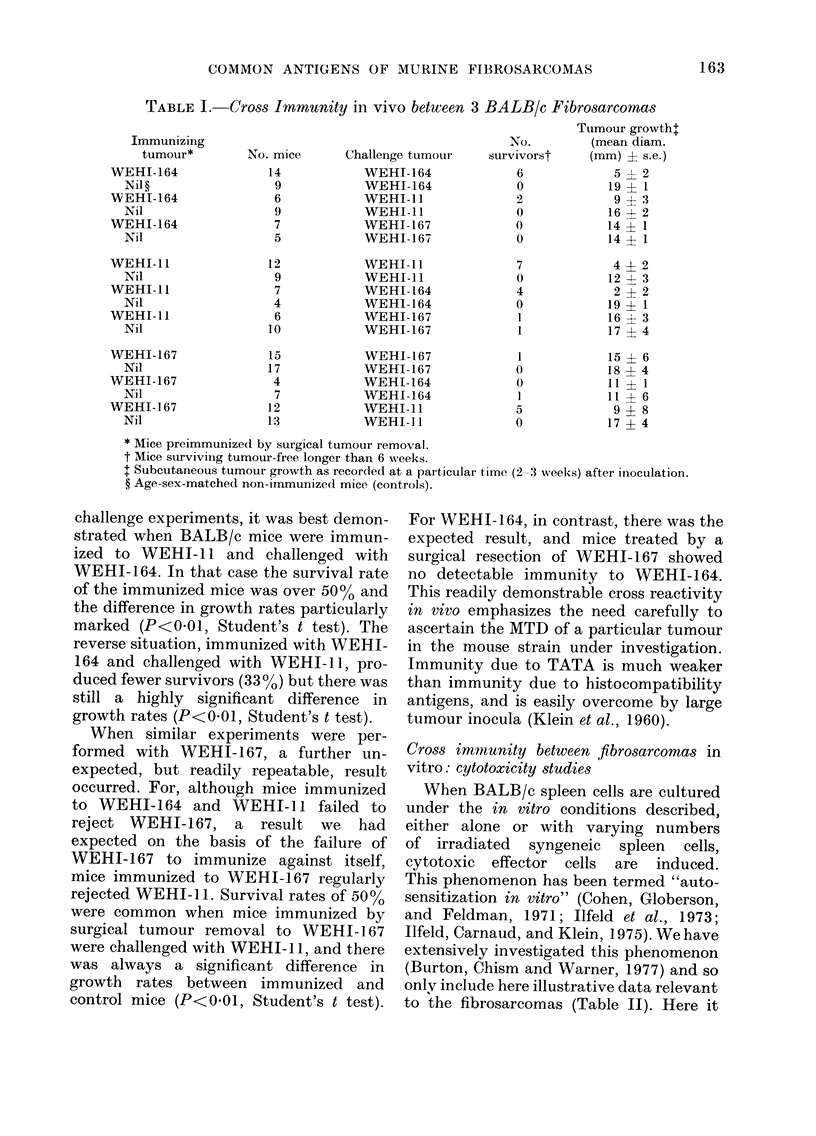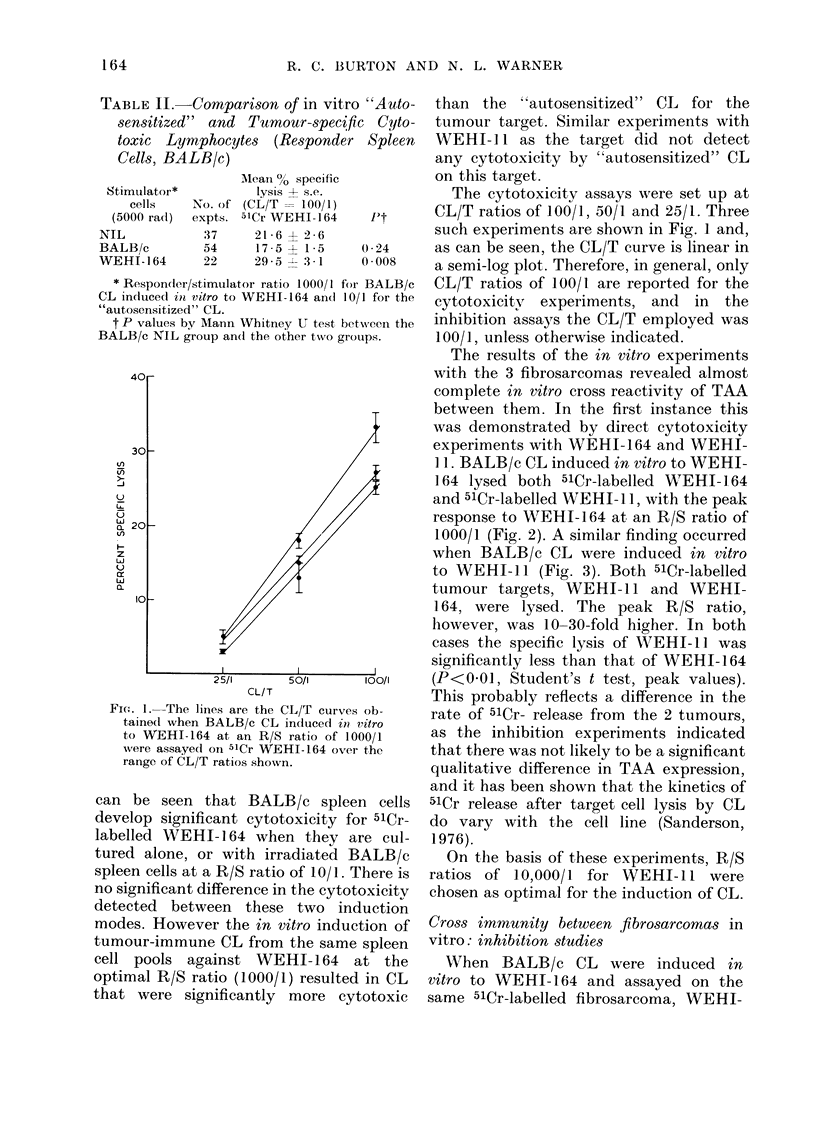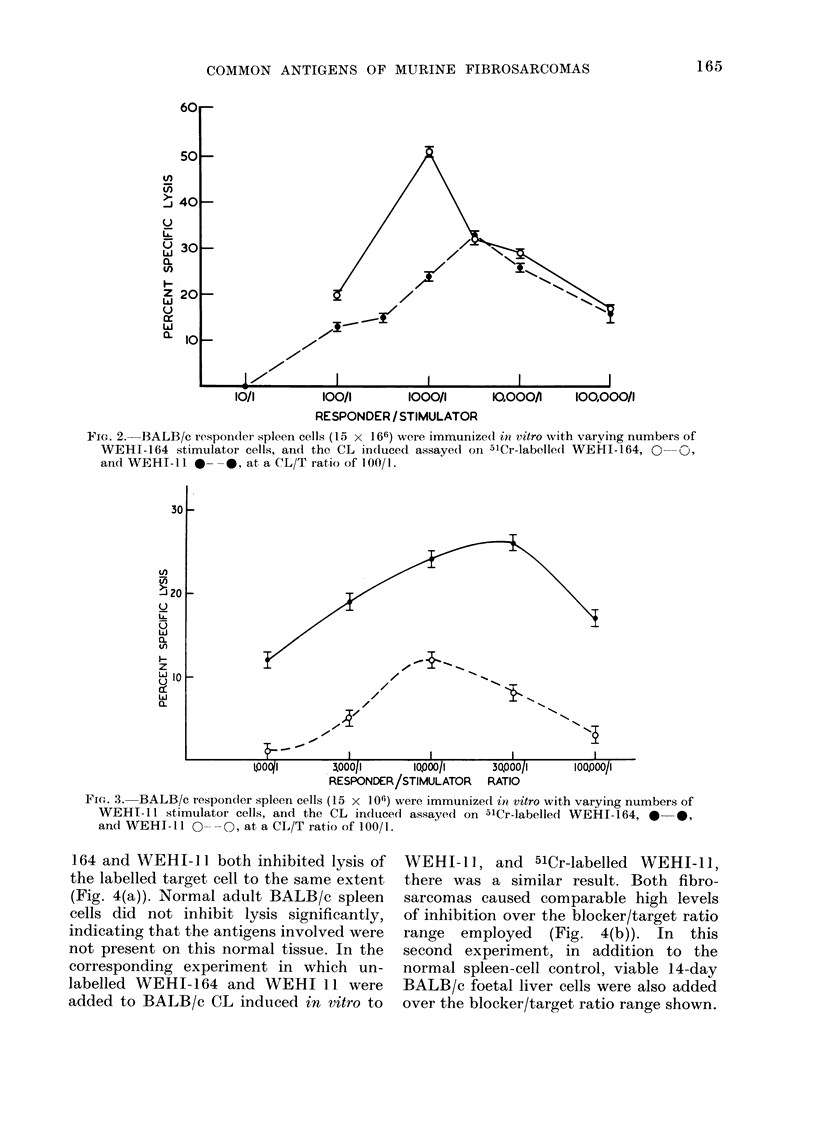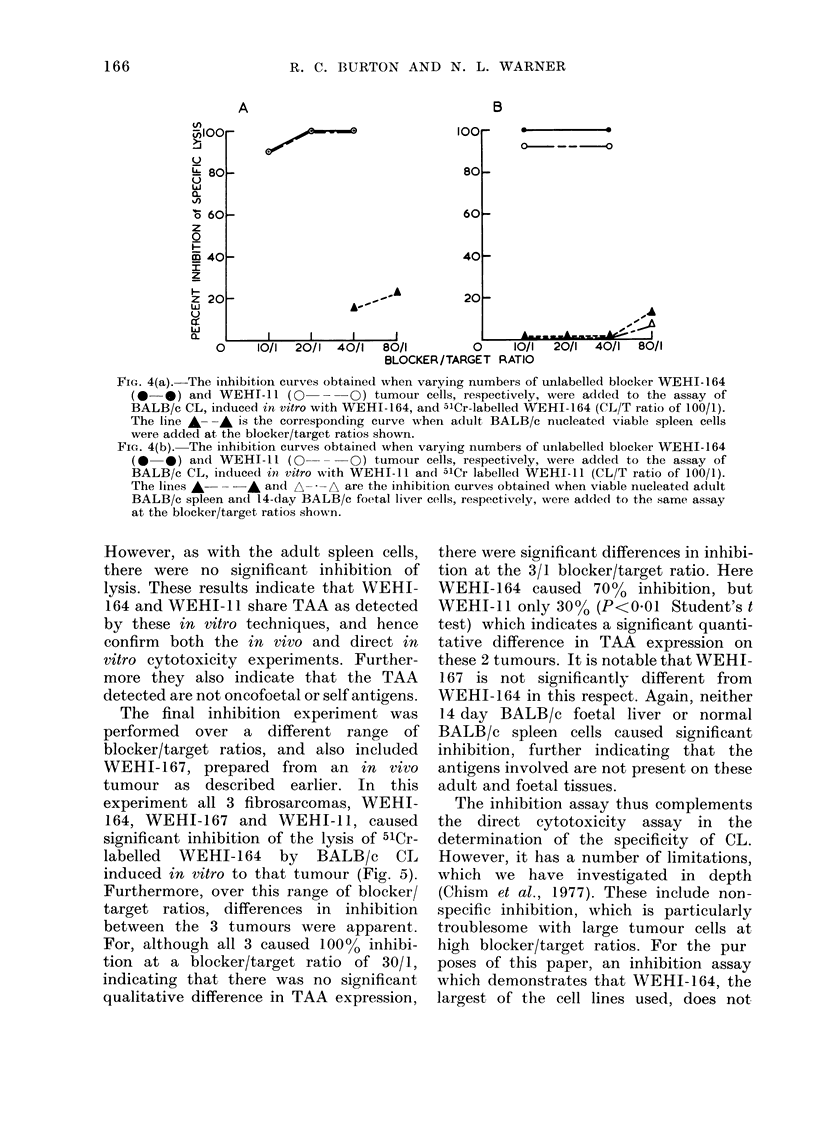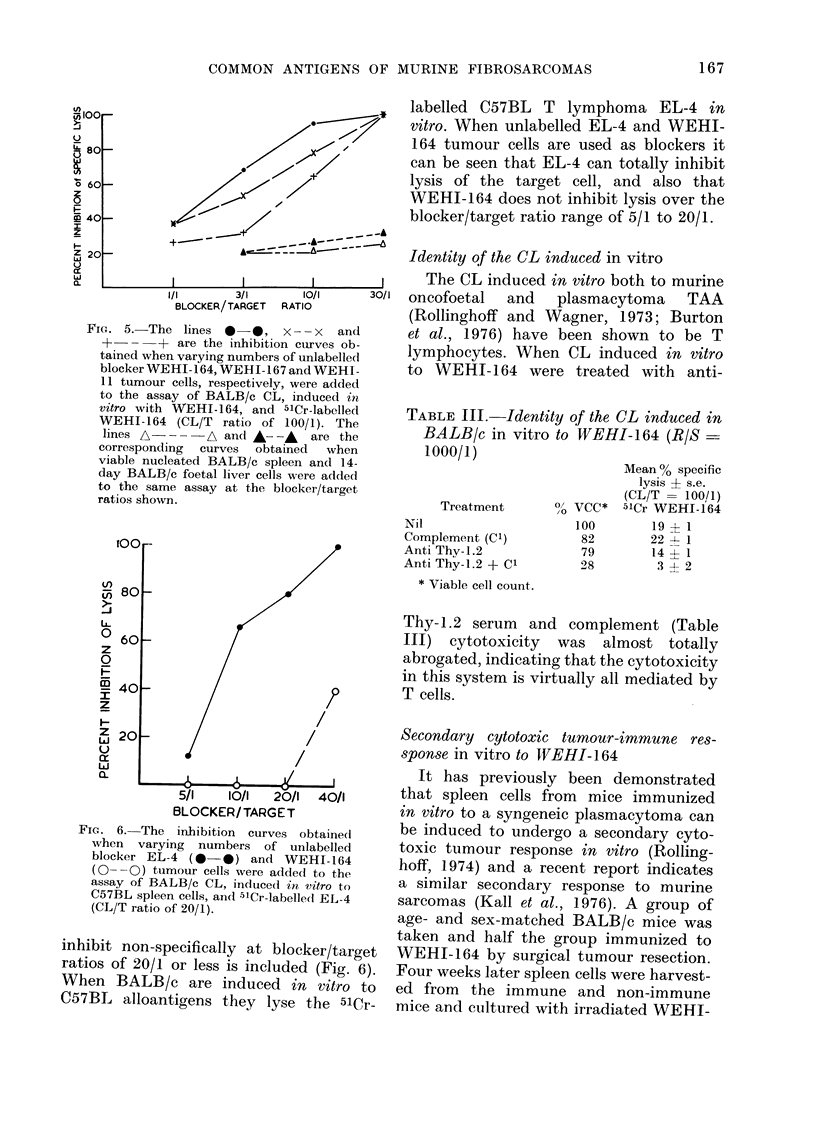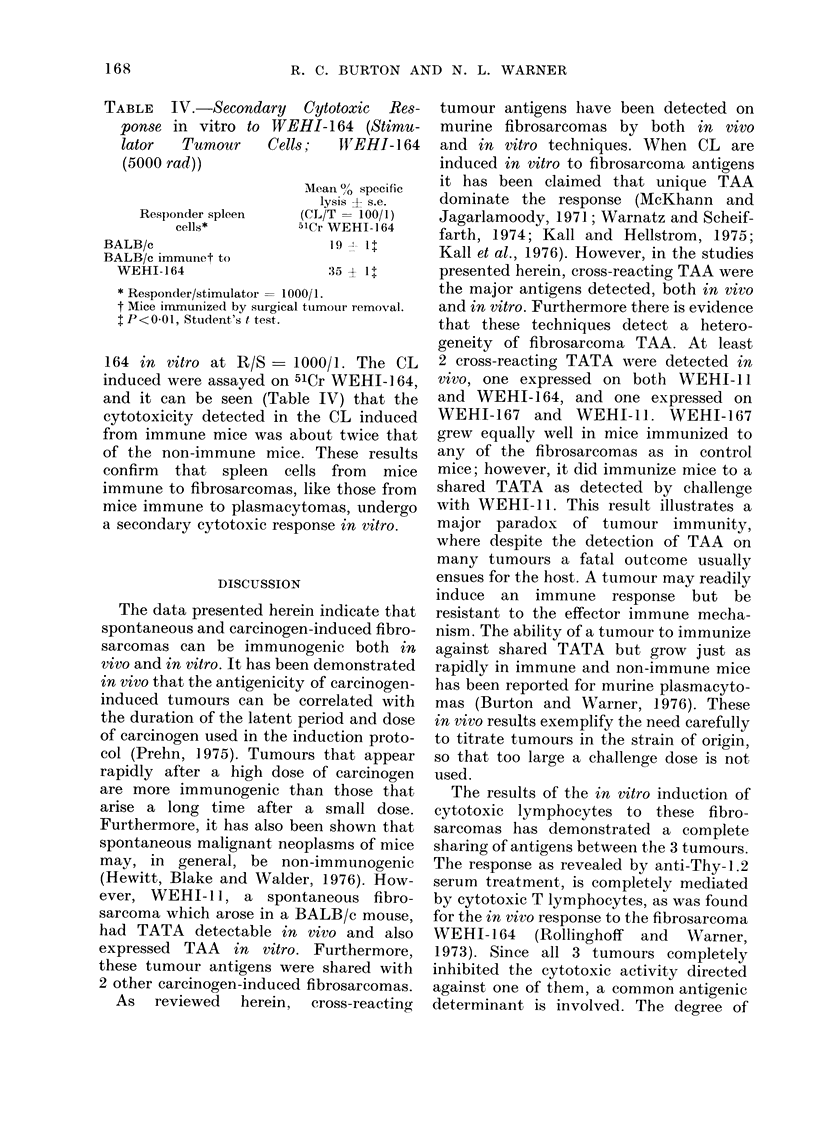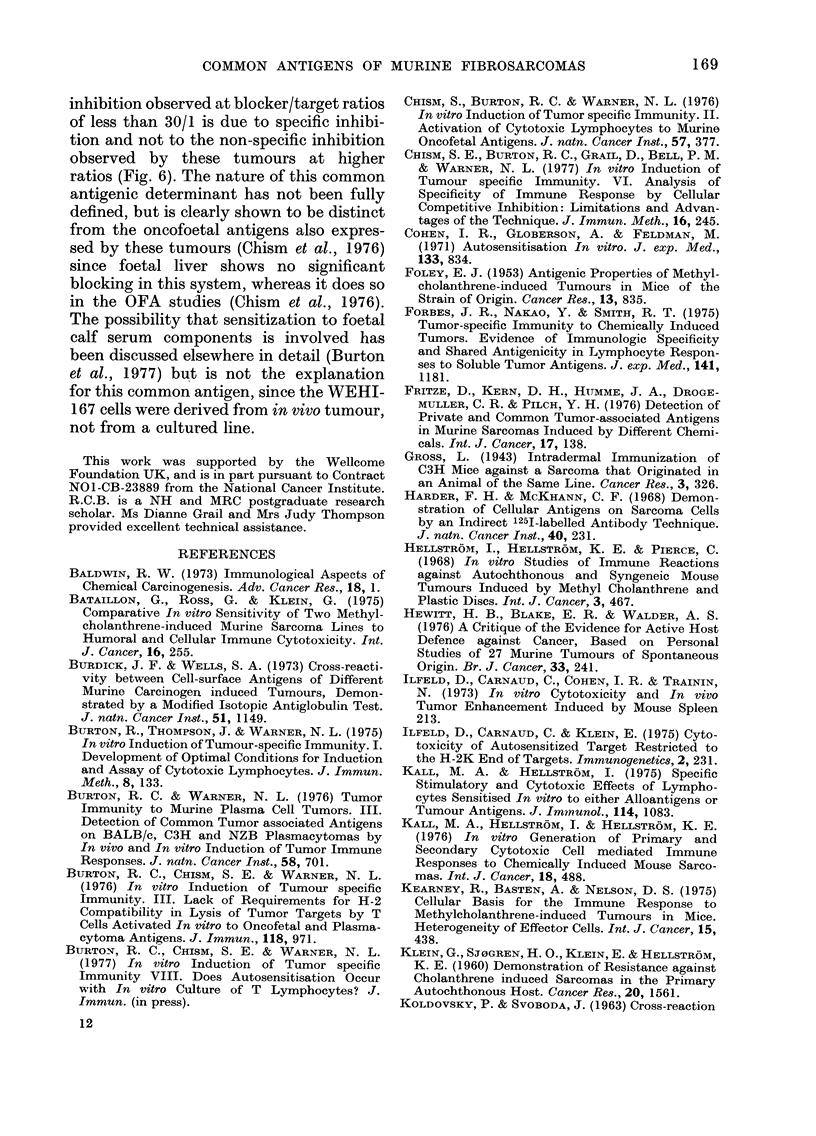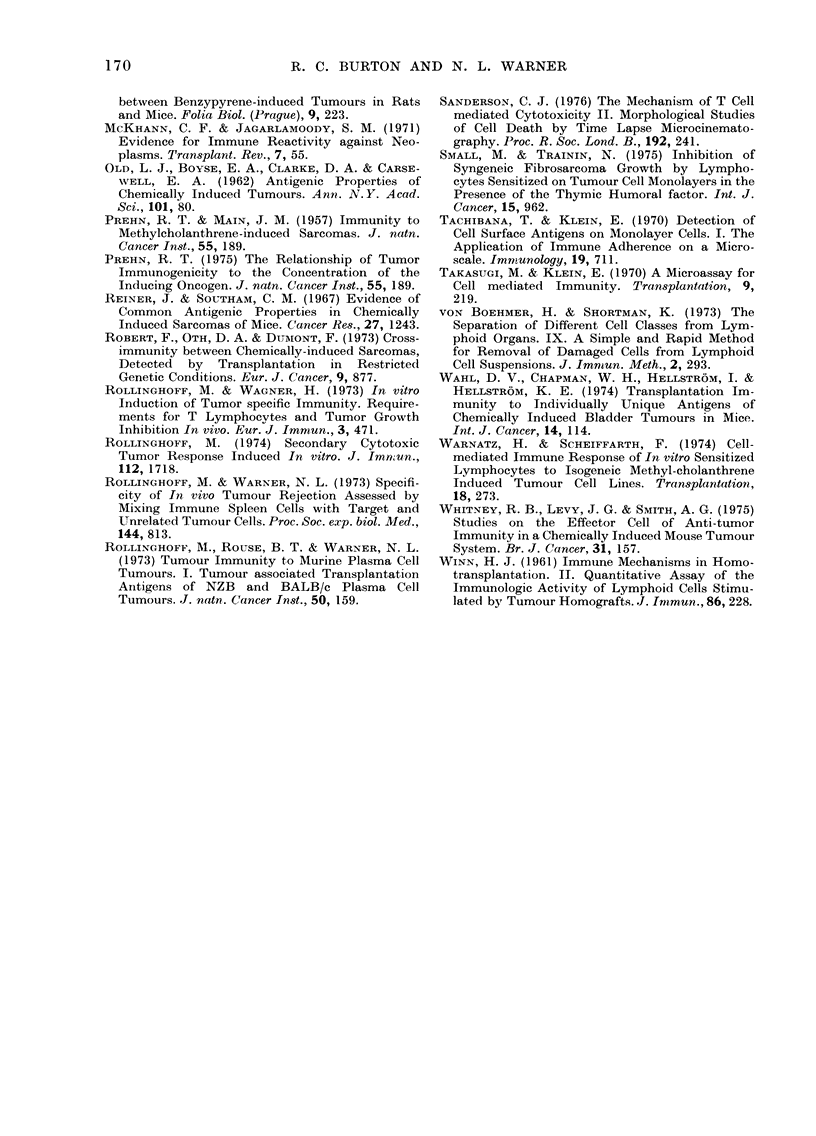# In vitro induction of tumour-specific immunity V. Detection of common antigenic determinatnts of murine fibrosarcomas.

**DOI:** 10.1038/bjc.1978.24

**Published:** 1978-02

**Authors:** R. C. Burton, N. L. Warner

## Abstract

Two 3-methylcholanthrene and a spontaneous BALB/c fibrosarcoma were examined for tumour-associated antigens (TAA) by in vivo and in vitro induction of tumour-immune responses. When BALB/c mice were immunized to these fibrosarcomas by surgical tumour removal, cross-reacting tumour-associated transplantation antigens (TATA) were detected on all 3 tumours. Cytotoxic effector cells (CL) were then induced in vitro by co-culture of BALB/c spleen cells with the spontaneous, or one of the carcinogen-induced fibrosarcomas. These CL were shown to be cytotoxic T cells (Tc) and to be directed against cross-reacting TAA on all 3 tumours, by two in vitro 51Cr-release assay systems, direct 51Cr-release cytotoxicity and cellular competitive inhibition of 51Cr release. Further studies demonstrated that the fibrosarcoma TAA involved in in vitro induction of Tc were not present on normal adult or foetal tissues. A secondary cytotoxic response was also detected in vitro when spleen cells from mice immunized to a carcinogen-induced fibrosarcoma were tested. The patterns of cross-reactivity detected by the in vivo and primary in vitro tumour-immune responses suggested that the TAA detected in vivo (TATA) were not identical to the TAA detected in vitro.


*Br. J. Cancer* (1978) **37**, 159

### *IN VITRO* INDUCTION OF TUMOUR-SPECIFIC IMMUNITY V. DETECTION OF COMMON ANTIGENIC DETERMINANTS OF MURINE FIBROSARCOMAS

R. C. BURTON AND N. L. WARNER*

*From the Genetics Unit, The Walter and Eliza Hall Institute of Medical Research, Melbourne, Australia*

Received 12 July 1977 Accepted 20 September 1977

**Summary.—Two 3-methylcholanthrene and a spontaneous BALB/c fibrosarcoma were examined for tumour-associated antigens (TAA) by *in vivo* and *in vitro* induction of tumour-immune responses. When BALB/c mice were immunized to these fibrosarcomas by surgical tumour removal, cross-reacting tumour-associated transplantation antigens (TATA) were detected on all 3 tumours. Cytotoxic effector cells (CL) were then induced *in vitro* by co-culture of BALB/c spleen cells with the spontaneous, or one of the carcinogen-induced fibrosarcomas. These CL were shown to be cytotoxic T cells (Tc) and to be directed against cross-reacting TAA on all 3 tumours, by two *in vitro* $^{51}$Cr-release assay systems, direct $^{51}$Cr-release cytotoxicity and cellular competitive inhibition of $^{51}$Cr release. Further studies demonstrated that the fibrosarcoma TAA involved in *in vitro* induction of Tc were not present on normal adult or foetal tissues. A secondary cytotoxic response was also detected *in vitro* when spleen cells from mice immunized to a carcinogen-induced fibrosarcoma were tested. The patterns of cross-reactivity detected by the *in vivo* and primary *in vitro* tumour-immune responses suggested that the TAA detected *in vivo* (TATA) were not identical to the TAA detected *in vitro*.**

THE first conclusive demonstration that tumours expressed specific tumour-associated antigens (TAA) was performed by Gross in 1943, using a 3 methylcholanthrene induced C3H murine sarcoma. Subsequent experimentation in highly inbred laboratory animals has firmly established the concept of TAA and, in particular, of "unique" tumour-associated transplantation antigens (TATA) on chemically induced tumours (Foley, 1953; Prehn and Main, 1957; Klein *et al.*, 1960; Old *et al.*, 1962). *In vivo* studies with chemically induced tumours in the ensuing years have generally confirmed these observations (Baldwin, 1973; Wahl *et al.*, 1974; Forbes, Nakao and Smith, 1975; Fritze *et al.*, 1976). However, in a few studies, shared TATA on chemically induced tumours have also been demonstrated (Koldovsky and Svoboda, 1963; Reiner and Southam, 1967; Robert, Oth and Dumont, 1973).

The introduction of *in vitro* methods for the study of TAA has, however, revealed a widespread sharing of TAA among chemically induced tumours. Cross-reacting TAA have been detected *in vitro* both by serological techniques (Harder and McKhann, 1968; Hellstrom, Hellstrom and Pierce, 1968; Tachibana and Klein, 1970; Burdick and Wells, 1973; Fritze *et al.*, 1976) and by assays of cell-mediated immunity where lymphoid cells from tumour-bearing or tumour-immune hosts were tested against various tumour cell lines (Hellstrom *et al.*, 1968; Takasugi and Klein, 1970; Bataillon, Ross and Klein, 1975; Forbes *et al.*, 1975; Whitney, Levy and Smith, 1975).

* Present address and address for reprints: University of New Mexico, School of Medicine, Department of Pathology, Albuquerque, NM 87131.

The *in vivo* specificity of immunity to chemically induced tumours has been ascribed to the immunization of T lymphocytes by "unique" TATA (Rollinghoff and Warner, 1973; Kearney, Basten and Nelson, 1975; Whitney *et al.*, 1975). The *in vitro* cross-reactivity of immunity, on the other hand, has been shown to vary both with the source of the effector cell (Bataillon *et al.*, 1975) and with the timing of cell harvest after immunization (Kearney *et al.*, 1975). The introduction of wholly *in vitro* methods for the induction *and* assay of tumour-specific immunity has provided another means of examining the TAA antigens of chemically induced tumours. McKhann and Jagarlamoody reported in 1971 the successful *in vitro* immunization of C3H spleen cells against 2 C3H 3-methylcholanthrene-induced fibrosarcomas. The immune lymphocytes (CL) were assayed both *in vitro*, on $^3$H-thymidine-labelled fibrosarcoma cells, and also *in vivo* in Winn-type assays (Klein *et al.*, 1960; Winn, 1961) where CL admixed with tumour cells were inoculated into syngeneic mice. The *in vivo* Winn assay results showed clear-cut specificity of *in vitro* immunization to "unique" TAA on each of the tumours, but the *in vitro* $^3$H-thymidine-release assay with the same CL demonstrated some cross-reactivity between the 2 fibrosarcomas. Subsequent studies have confirmed the *in vitro* immunization of lymphocytes by TAA on carcinogen-induced tumours (Warnatz and Scheiffarth, 1974; Small and Trainin, 1975; Kall and Hellstrom, 1975). However, conflicting results have emerged from these *in vitro* experiments, with claims both that the specificity of the *in vitro* tumour specific immunity induced is to unique TAA alone (McKhann and Jagarlamoody, 1971; Kall, Hellstrom and Hellstrom, 1976) and that it is to both cross-reacting and unique TAA on the same tumour cells (Warnatz and Scheiffarth, 1974). The identity of the effector cytotoxic cells in these *in vitro* systems was not described.

This paper presents our experience with both *in vitro* and *in vivo* induction of tumour-specific immunity to 3 BALB/c fibrosarcomas, with particular reference to the TAA involved. Also included is data which indicates that the *in vitro* cytotoxic cell is a T lymphocyte.

### MATERIALS AND METHODS

*Media.*—Eagle's minimal essential medium with non-essential amino acids (Grand Island Biological Co., NY) was used supplemented with 10% foetal calf serum (FCS) (Commonwealth Serum Laboratories) 100 u/ml of penicillin, 100 $\mu$g/ml of streptomycin and buffered with sodium bicarbonate (MEMF). The medium was prepared fresh each day, and 2-mercaptoethanol (2ME) was added to a final concentration of $10^{-4}$M. The assay was performed in Dulbecco's modified Eagle's medium (Commonwealth Serum Laboratories, Melbourne, Australia) supplemented with 10% FCS (DMEF).

*Animals.*—Inbred male mice, aged 6–10 weeks, from the Hall Institute BALB/c An/Bradley/WEHI specific-pathogen-free colony, were used throughout this study.

*Tumours.*—Three BALB/c fibrosarcomas were used in this study, and were adapted to tissue culture early in their transplantation history. WEHI-164 and WEHI-167 are 3-methylcholanthrene-induced BALB/c fibrosarcomas whose origins and adaptation to tissue culture have been described previously (Rollinghoff and Warner, 1973). WEHI-11 is a BALB/c fibrosarcoma which arose spontaneously, and was adapted to tissue culture by a technique described previously (Rollinghoff and Warner, 1973). The tissue-culture lines of WEHI-164 and WEHI-11 were used for all the *in vitro* experiments involving these 2 tumours, whereas in the *in vivo* experiments the immunizations were performed with *in vivo*-derived cells, and the tumour challenge with either *in vivo*- or *in vitro*-derived cells. WEHI-167, however, proved difficult to maintain in tissue culture, and was therefore inoculated into BALB/c mice and maintained in serial passage *in vivo*. The WEHI-167 tumour cells used in this study were derived from cell suspensions prepared from *in vivo* tumours according to the method of von Boehmer and Shortman (1973). This technique removes dead cells and cellular debris from the suspension, leaving a single-cell tumour suspension of over 80% viability as determined by eosin dye exclusion. EL-4, a C57BL T lymphoma maintained in tissue culture, was used for the demonstration of specificity in the inhibition assay, and was kindly provided by Dr Alan Harris.

In vivo *immunization*.—The 3 fibrosarcomas were tested for the presence of TATA in BALB/c mice by the technique of surgical tumour removal as previously described (Rollinghoff, Rouse and Warner, 1973; Burton and Warner, 1976). Briefly, the minimum tumour dose (MTD) of viable tumour cells capable of initiating lethal tumour growth in 100% of inoculated BALB/c mice was determined for each tumour. Groups of BALB/c mice were then injected in the right hind leg with a dose in excess of this, and the legs amputated under ether anaesthesia when the tumour became palpable. Two weeks later these mice were challenged, together with age- and sex-matched BALB/c controls, s.c. on the right flank with the MTD of the immunizing tumour. The mice were then observed 2–3 times weekly and the tumour diameters determined by taking the mean of 2 transverse measurements of the tumour mass.

In the cross-immunity experiments, twice as many BALB/c mice were immunized by this procedure, and half challenged with the MTD of the immunizing tumour, and half with the MTD of the tumour being tested for cross immunity.

In vitro *induction and assay of tumour-specific immunity*.—The techniques have been previously described in detail, and so are only outlined here.

*Induction procedure* (Burton, Thompson and Warner, 1975).—Square 100mm tissue-culture trays partitioned into 25 compartments (Sterilin Ltd, Richmond, Surrey, England) were used. Varying numbers of irradiated (5000 rad) stimulator fibrosarcoma cells, in a volume of 0·1 ml, were placed into the compartments and 3·6 ml of MEMF added. Then $15 \times 10^6$ viable nucleated responder BALB/c spleen cells (in 0·2 ml) were added to each compartment, and the trays placed in a humidified incubator at 37°C in 10% $CO_2$ in air for 5 days; these conditions being optimal for the induction of tumour-specific immunity. This method was adopted in order to produce responder/stimulator curves for the various *in vitro* direct cytotoxicity experiments. Groups of 10 identical compartments were set up for each ratio, and stimulator cell numbers, in the range of $1{\cdot}5 \times 10^2$–$1{\cdot}5 \times 10^5$ per compartment, were used. In addition, irradiated (5000 rad) C57BL stimulator spleen cells were used to alloimmunize BALB/c responder lymphocytes *in vitro* by culturing the 2 cell types together as described above, but at a responder/stimulator (R/S) ratio of 10/1. BALB/c responder spleen cells alone or with $1{\cdot}5 \times 10^6$ irradiated (5000 rad) syngeneic stimulator BALB/c spleen cells.

At the end of the incubation period the cultured cells were harvested, pelleted by light centrifugation, identical CL lots pooled, and resuspended in fresh DMEF. They were then further incubated in 35mm Petri dishes for 21 h, as this results in a 2–3-fold augmentation of lytic activity. Finally they were harvested and a viable cytotoxic effector cell (CL) count was performed by eosin dye exclusion.

*Direct* $^{51}$*Cr-release cytotoxic assay* (Burton *et al.*, 1975).—This was performed in quadruplicate in microtitre trays (Micro Test II Tissue Culture Plate, Falcon Plastics, Oxnard, California). Fibrosarcoma tissue-culture cells were labelled with $^{51}Cr$. Background release of $^{51}Cr$ was determined by incubating $25 \times 10^3$ of these labelled tumour target cells in 200 $\mu$l of DMEF. The maximal amount of $^{51}Cr$ releasable from the targets was assessed by lysing aliquots of $12{\cdot}5 \times 10^3$ labelled target cells in 200 $\mu$l of Zaponin (improved lysory agent for white blood cell counts, Coulter Electronics Ltd, Dunstable, Beds., UK) in distilled water. The test assays were made at CL/target (CL/T) ratios of 100:1, 50:1 and 25:1. Thus $25 \times 10^5$, $12{\cdot}5 \times 10^5$ and $6{\cdot}25 \times 10^6$ CL in 100 $\mu$l of DMEF were dispensed into wells and then $25 \times 10^3$ labelled tumour target cells in 100 $\mu$l of DMEF were added.

The standard assay time was 4 h at 37°C followed by an additional hour at 45°C to facilitate the release of $^{51}Cr$ from the lysed tumour target cells. Then 100 $\mu$l of supernatant was removed from each of the background and test assay wells, while from the Zaponin dilution wells 100$\mu$l aliquots from 2 wells were pooled, since each well contained half the number of targets. These samples were counted on a Beckman Biogamma scintillation counter, and the percent specific lysis computed as

Percentage specific lysis=

$$\frac{\text{test count} - \text{background count}}{\text{maximal count} - \text{background count}} \times 100$$

*Inhibition assay* (Chism, Burton and Warner, 1976).—The CL were first added ($25 \times 10^5$ in 50 $\mu$l) to the test well of the microtitre tray, followed by varying numbers (in 50 $\mu$l) of unlabelled inhibitor cell preparations (blocker cells) and then the $^{51}$Cr-labelled tumour target cells ($25 \times 10^3$ in 100 $\mu$l). The assay conditions were then as described above for direct lysis. Control tubes assessed specific lysis in the absence of blocker cells, and the results were expressed as percent inhibition of specific lysis. In all these studies the absolute number of CL and $^{51}$Cr target cells remained constant, only the blocker-cell number was varied, and this is given in the test figures as a blocker/target cell ratio.

Percentage inhibition of specific lysis=

$$\frac{\text{control lysis} - \text{test lysis}}{\text{control lysis}} \times 100$$

Blocker cells included the 3 fibrosarcomas, EL-4, adult BALB/c viable nucleated spleen cells, and 14-day viable nucleated BALB/c foetal liver cells. The foetal-cell suspension was prepared according to a technique described previously (Chism *et al.*, 1976).

*Anti-Thy-1.2 treatment.*—Anti-Thy-1.2 sera was prepared and supplied by Ms J. Gamble. Its mode of production, cytotoxic titre and specificity for T cells have been described in detail (Burton, Chism and Warner, 1976). *In vitro*-induced CL were treated with the serum at a dilution of 1:3 for 30 min at 37°C, after which they were resuspended in a 1:6 dilution of agarose-absorbed guinea-pig complement for a further 30 min at 37°C. The CL were then counted and made up to $25 + 10^6$/ml in DMEF for the assay.

% VCC=

$$\frac{\text{total viable CL after treatment}}{\text{total viable CL before treatment}} \times 100$$

### RESULTS

### *Cross immunity between fibrosarcomas* in vivo

The technique of surgical tumour resection proved a reliable method for inducing tumour immunity *in vivo* (Table I). The use of the MTD for the challenge dose of a particular tumour allowed a clear demonstration of immunity to that tumour. Each particular tumour immunization and challenge combination was performed at least 3 times, and the results presented are from representative experiments. The first 2 lines of each section of the Table show the result of attempts to demonstrate TATA on the 3 fibrosarcomas. Both WEHI-164 and WEHI-11 regularly elicited tumour-specific immunity in the strain of origin, and in both cases there was a significant difference in the growth rate between the immunized and control mice when both groups were challenged with the MTD of the immunizing tumour ($P < 0{\cdot}01$, Student's $t$ test). Furthermore, $\sim$ 50% of the mice in the groups immunized by surgical tumour removal to either of these tumours survived a challenge with the MTD of the relevant tumour, while there were no survivors in the control groups. WEHI-167, on the other hand, did not effectively immunize BALB/c mice against itself. There was no difference in the growth rate of this tumour between the mice who underwent a surgical tumour removal of WEHI-167 and the control mice, in any of the 5 experiments. In the example shown there was one survivor in the treated group, and the general finding was that survivors also were rare when mice who had undergone resection of WEHI-167 were challenged with the MTD of the tumour. Mice immunized with WEHI-164 cells do not show any significant degree of immunity to challenge with a tissue culture line of a plasmacytoma (MPC-11) indicating that cell-bound foetal calf serum antigens are probably not involved in the immunity demonstrated in Table I.

The cross-immunity experiments, however, produced some unexpected findings. In the first instance, there was strong cross immunity between WEHI-11 and WEHI-164, indicating a highly immunogenic shared TATA. Although readily detectable in reciprocal immunization and

TABLE I.—*Cross Immunity* in vivo *between 3 BALB/c Fibrosarcomas*

| Immunizing tumour* | No. mice | Challenge tumour | No. survivors† | Tumour growth‡ (mean diam. (mm) ± s.e.) |
|---|---|---|---|---|
| WEHI-164 | 14 | WEHI-164 | 6 | 5 ± 2 |
| Nil§ | 9 | WEHI-164 | 0 | 19 ± 1 |
| WEHI-164 | 6 | WEHI-11 | 2 | 9 ± 3 |
| Nil | 9 | WEHI-11 | 0 | 16 ± 2 |
| WEHI-164 | 7 | WEHI-167 | 0 | 14 ± 1 |
| Nil | 5 | WEHI-167 | 0 | 14 ± 1 |
| WEHI-11 | 12 | WEHI-11 | 7 | 4 ± 2 |
| Nil | 9 | WEHI-11 | 0 | 12 ± 3 |
| WEHI-11 | 7 | WEHI-164 | 4 | 2 ± 2 |
| Nil | 4 | WEHI-164 | 0 | 19 ± 1 |
| WEHI-11 | 6 | WEHI-167 | 1 | 16 ± 3 |
| Nil | 10 | WEHI-167 | 1 | 17 ± 4 |
| WEHI-167 | 15 | WEHI-167 | 1 | 15 ± 6 |
| Nil | 17 | WEHI-167 | 0 | 18 ± 4 |
| WEHI-167 | 4 | WEHI-164 | 0 | 11 ± 1 |
| Nil | 7 | WEHI-164 | 1 | 11 ± 6 |
| WEHI-167 | 12 | WEHI-11 | 5 | 9 ± 8 |
| Nil | 13 | WEHI-11 | 0 | 17 ± 4 |

* Mice preimmunized by surgical tumour removal.
† Mice surviving tumour-free longer than 6 weeks.
‡ Subcutaneous tumour growth as recorded at a particular time (2–3 weeks) after inoculation.
§ Age-sex-matched non-immunized mice (controls).

challenge experiments, it was best demonstrated when BALB/c mice were immunized to WEHI-11 and challenged with WEHI-164. In that case the survival rate of the immunized mice was over 50% and the difference in growth rates particularly marked ($P<0 \cdot 01$, Student's $t$ test). The reverse situation, immunized with WEHI-164 and challenged with WEHI-11, produced fewer survivors (33%) but there was still a highly significant difference in growth rates ($P<0 \cdot 01$, Student's $t$ test).

When similar experiments were performed with WEHI-167, a further unexpected, but readily repeatable, result occurred. For, although mice immunized to WEHI-164 and WEHI-11 failed to reject WEHI-167, a result we had expected on the basis of the failure of WEHI-167 to immunize against itself, mice immunized to WEHI-167 regularly rejected WEHI-11. Survival rates of 50% were common when mice immunized by surgical tumour removal to WEHI-167 were challenged with WEHI-11, and there was always a significant difference in growth rates between immunized and control mice ($P<0 \cdot 01$, Student's $t$ test).

For WEHI-164, in contrast, there was the expected result, and mice treated by a surgical resection of WEHI-167 showed no detectable immunity to WEHI-164. This readily demonstrable cross reactivity *in vivo* emphasizes the need carefully to ascertain the MTD of a particular tumour in the mouse strain under investigation. Immunity due to TATA is much weaker than immunity due to histocompatibility antigens, and is easily overcome by large tumour inocula (Klein *et al.*, 1960).

*Cross immunity between fibrosarcomas* in vitro*: cytotoxicity studies*

When BALB/c spleen cells are cultured under the *in vitro* conditions described, either alone or with varying numbers of irradiated syngeneic spleen cells, cytotoxic effector cells are induced. This phenomenon has been termed "autosensitization *in vitro*" (Cohen, Globerson, and Feldman, 1971; Ilfeld *et al.*, 1973; Ilfeld, Carnaud, and Klein, 1975). We have extensively investigated this phenomenon (Burton, Chism and Warner, 1977) and so only include here illustrative data relevant to the fibrosarcomas (Table II). Here it

TABLE II.—*Comparison of* in vitro *"Autosensitized" and Tumour-specific Cytotoxic Lymphocytes (Responder Spleen Cells, BALB/c)*

| Stimulator* cells (5000 rad) | No. of expts. | Mean % specific lysis ± s.e. (CL/T = 100/1) $^{51}$Cr WEHI-164 | $P$† |
|---|---|---|---|
| NIL | 37 | 21·6 ± 2·6 | |
| BALB/c | 54 | 17·5 ± 1·5 | 0·24 |
| WEHI-164 | 22 | 29·5 ± 3·1 | 0·008 |

* Responder/stimulator ratio 1000/1 for BALB/c CL induced *in vitro* to WEHI-164 and 10/1 for the "autosensitized" CL.

† $P$ values by Mann Whitney U test between the BALB/c NIL group and the other two groups.

FIG. 1.—The lines are the CL/T curves obtained when BALB/c CL induced *in vitro* to WEHI-164 at an R/S ratio of 1000/1 were assayed on $^{51}$Cr WEHI-164 over the range of CL/T ratios shown.

can be seen that BALB/c spleen cells develop significant cytotoxicity for $^{51}$Cr-labelled WEHI-164 when they are cultured alone, or with irradiated BALB/c spleen cells at a R/S ratio of 10/1. There is no significant difference in the cytotoxicity detected between these two induction modes. However the *in vitro* induction of tumour-immune CL from the same spleen cell pools against WEHI-164 at the optimal R/S ratio (1000/1) resulted in CL that were significantly more cytotoxic than the "autosensitized" CL for the tumour target. Similar experiments with WEHI-11 as the target did not detect any cytotoxicity by "autosensitized" CL on this target.

The cytotoxicity assays were set up at CL/T ratios of 100/1, 50/1 and 25/1. Three such experiments are shown in Fig. 1 and, as can be seen, the CL/T curve is linear in a semi-log plot. Therefore, in general, only CL/T ratios of 100/1 are reported for the cytotoxicity experiments, and in the inhibition assays the CL/T employed was 100/1, unless otherwise indicated.

The results of the *in vitro* experiments with the 3 fibrosarcomas revealed almost complete *in vitro* cross reactivity of TAA between them. In the first instance this was demonstrated by direct cytotoxicity experiments with WEHI-164 and WEHI-11. BALB/c CL induced *in vitro* to WEHI-164 lysed both $^{51}$Cr-labelled WEHI-164 and $^{51}$Cr-labelled WEHI-11, with the peak response to WEHI-164 at an R/S ratio of 1000/1 (Fig. 2). A similar finding occurred when BALB/c CL were induced *in vitro* to WEHI-11 (Fig. 3). Both $^{51}$Cr-labelled tumour targets, WEHI-11 and WEHI-164, were lysed. The peak R/S ratio, however, was 10–30-fold higher. In both cases the specific lysis of WEHI-11 was significantly less than that of WEHI-164 ($P<0{\cdot}01$, Student's $t$ test, peak values). This probably reflects a difference in the rate of $^{51}$Cr- release from the 2 tumours, as the inhibition experiments indicated that there was not likely to be a significant qualitative difference in TAA expression, and it has been shown that the kinetics of $^{51}$Cr release after target cell lysis by CL do vary with the cell line (Sanderson, 1976).

On the basis of these experiments, R/S ratios of 10,000/1 for WEHI-11 were chosen as optimal for the induction of CL.

*Cross immunity between fibrosarcomas* in vitro*: inhibition studies*

When BALB/c CL were induced *in vitro* to WEHI-164 and assayed on the same $^{51}$Cr-labelled fibrosarcoma, WEHI-

FIG. 2.—BALB/c responder spleen cells (15 × 16[6]) were immunized *in vitro* with varying numbers of WEHI-164 stimulator cells, and the CL induced assayed on $^{51}$Cr-labelled WEHI-164, ○—○, and WEHI-11 ●- -●, at a CL/T ratio of 100/1.

FIG. 3.—BALB/c responder spleen cells (15 × 10[6]) were immunized *in vitro* with varying numbers of WEHI-11 stimulator cells, and the CL induced assayed on $^{51}$Cr-labelled WEHI-164, ●—●, and WEHI-11 ○- -○, at a CL/T ratio of 100/1.

164 and WEHI-11 both inhibited lysis of the labelled target cell to the same extent (Fig. 4(a)). Normal adult BALB/c spleen cells did not inhibit lysis significantly, indicating that the antigens involved were not present on this normal tissue. In the corresponding experiment in which unlabelled WEHI-164 and WEHI 11 were added to BALB/c CL induced *in vitro* to WEHI-11, and $^{51}$Cr-labelled WEHI-11, there was a similar result. Both fibrosarcomas caused comparable high levels of inhibition over the blocker/target ratio range employed (Fig. 4(b)). In this second experiment, in addition to the normal spleen-cell control, viable 14-day BALB/c foetal liver cells were also added over the blocker/target ratio range shown.

FIG. 4(a).—The inhibition curves obtained when varying numbers of unlabelled blocker WEHI-164 (●—●) and WEHI-11 (○– – –○) tumour cells, respectively, were added to the assay of BALB/c CL, induced *in vitro* with WEHI-164, and $^{51}$Cr-labelled WEHI-164 (CL/T ratio of 100/1). The line ▲– –▲ is the corresponding curve when adult BALB/c nucleated viable spleen cells were added at the blocker/target ratios shown.

FIG. 4(b).—The inhibition curves obtained when varying numbers of unlabelled blocker WEHI-164 (●—●) and WEHI-11 (○– – –○) tumour cells, respectively, were added to the assay of BALB/c CL, induced *in vitro* with WEHI-11 and $^{51}$Cr labelled WEHI-11 (CL/T ratio of 100/1). The lines ▲– – –▲ and △–·–△ are the inhibition curves obtained when viable nucleated adult BALB/c spleen and 14-day BALB/c foetal liver cells, respectively, were added to the same assay at the blocker/target ratios shown.

However, as with the adult spleen cells, there were no significant inhibition of lysis. These results indicate that WEHI-164 and WEHI-11 share TAA as detected by these *in vitro* techniques, and hence confirm both the *in vivo* and direct *in vitro* cytotoxicity experiments. Furthermore they also indicate that the TAA detected are not oncofoetal or self antigens.

The final inhibition experiment was performed over a different range of blocker/target ratios, and also included WEHI-167, prepared from an *in vivo* tumour as described earlier. In this experiment all 3 fibrosarcomas, WEHI-164, WEHI-167 and WEHI-11, caused significant inhibition of the lysis of $^{51}$Cr-labelled WEHI-164 by BALB/c CL induced *in vitro* to that tumour (Fig. 5). Furthermore, over this range of blocker/target ratios, differences in inhibition between the 3 tumours were apparent. For, although all 3 caused 100% inhibition at a blocker/target ratio of 30/1, indicating that there was no significant qualitative difference in TAA expression, there were significant differences in inhibition at the 3/1 blocker/target ratio. Here WEHI-164 caused 70% inhibition, but WEHI-11 only 30% ($P<0\cdot01$ Student's $t$ test) which indicates a significant quantitative difference in TAA expression on these 2 tumours. It is notable that WEHI-167 is not significantly different from WEHI-164 in this respect. Again, neither 14 day BALB/c foetal liver or normal BALB/c spleen cells caused significant inhibition, further indicating that the antigens involved are not present on these adult and foetal tissues.

The inhibition assay thus complements the direct cytotoxicity assay in the determination of the specificity of CL. However, it has a number of limitations, which we have investigated in depth (Chism *et al.*, 1977). These include non-specific inhibition, which is particularly troublesome with large tumour cells at high blocker/target ratios. For the purposes of this paper, an inhibition assay which demonstrates that WEHI-164, the largest of the cell lines used, does not

FIG. 5.—The lines ●—●, ×- -× and +— — —+ are the inhibition curves obtained when varying numbers of unlabelled blocker WEHI-164, WEHI-167 and WEHI-11 tumour cells, respectively, were added to the assay of BALB/c CL, induced *in vitro* with WEHI-164, and $^{51}$Cr-labelled WEHI-164 (CL/T ratio of 100/1). The lines △— — — —△ and ▲- -▲ are the corresponding curves obtained when viable nucleated BALB/c spleen and 14-day BALB/c foetal liver cells were added to the same assay at the blocker/target ratios shown.

FIG. 6.—The inhibition curves obtained when varying numbers of unlabelled blocker EL-4 (●—●) and WEHI-164 (○- -○) tumour cells were added to the assay of BALB/c CL, induced *in vitro* to C57BL spleen cells, and $^{51}$Cr-labelled EL-4 (CL/T ratio of 20/1).

inhibit non-specifically at blocker/target ratios of 20/1 or less is included (Fig. 6). When BALB/c are induced *in vitro* to C57BL alloantigens they lyse the $^{51}$Cr-labelled C57BL T lymphoma EL-4 *in vitro*. When unlabelled EL-4 and WEHI-164 tumour cells are used as blockers it can be seen that EL-4 can totally inhibit lysis of the target cell, and also that WEHI-164 does not inhibit lysis over the blocker/target ratio range of 5/1 to 20/1.

### *Identity of the CL induced* in vitro

The CL induced *in vitro* both to murine oncofoetal and plasmacytoma TAA (Rollinghoff and Wagner, 1973; Burton *et al.*, 1976) have been shown to be T lymphocytes. When CL induced *in vitro* to WEHI-164 were treated with anti-Thy-1.2 serum and complement (Table III) cytotoxicity was almost totally abrogated, indicating that the cytotoxicity in this system is virtually all mediated by T cells.

TABLE III.—*Identity of the CL induced in BALB/c* in vitro *to WEHI*-164 (*R/S* = 1000/1)

| Treatment | % VCC* | Mean % specific lysis ± s.e. (CL/T = 100/1) $^{51}$Cr WEHI-164 |
|---|---|---|
| Nil | 100 | 19 ± 1 |
| Complement (C¹) | 82 | 22 ± 1 |
| Anti Thy-1.2 | 79 | 14 ± 1 |
| Anti Thy-1.2 + C¹ | 28 | 3 ± 2 |

* Viable cell count.

### *Secondary cytotoxic tumour-immune response* in vitro *to WEHI*-164

It has previously been demonstrated that spleen cells from mice immunized *in vitro* to a syngeneic plasmacytoma can be induced to undergo a secondary cytotoxic tumour response *in vitro* (Rollinghoff, 1974) and a recent report indicates a similar secondary response to murine sarcomas (Kall *et al.*, 1976). A group of age- and sex-matched BALB/c mice was taken and half the group immunized to WEHI-164 by surgical tumour resection. Four weeks later spleen cells were harvested from the immune and non-immune mice and cultured with irradiated WEHI-

TABLE IV.—*Secondary Cytotoxic Response* in vitro *to WEHI*-164 (*Stimulator Tumour Cells; WEHI*-164 (5000 *rad*))

| Responder spleen cells* | Mean % specific lysis ± s.e. (CL/T = 100/1) $^{51}Cr$ WEHI-164 |
|---|---|
| BALB/c | 19 ± 1‡ |
| BALB/c immune† to WEHI-164 | 35 ± 1‡ |

* Responder/stimulator = 1000/1.
† Mice immunized by surgical tumour removal.
‡ $P<0.01$, Student's $t$ test.

164 *in vitro* at R/S = 1000/1. The CL induced were assayed on $^{51}Cr$ WEHI-164, and it can be seen (Table IV) that the cytotoxicity detected in the CL induced from immune mice was about twice that of the non-immune mice. These results confirm that spleen cells from mice immune to fibrosarcomas, like those from mice immune to plasmacytomas, undergo a secondary cytotoxic response *in vitro*.

### DISCUSSION

The data presented herein indicate that spontaneous and carcinogen-induced fibrosarcomas can be immunogenic both *in vivo* and *in vitro*. It has been demonstrated *in vivo* that the antigenicity of carcinogen-induced tumours can be correlated with the duration of the latent period and dose of carcinogen used in the induction protocol (Prehn, 1975). Tumours that appear rapidly after a high dose of carcinogen are more immunogenic than those that arise a long time after a small dose. Furthermore, it has also been shown that spontaneous malignant neoplasms of mice may, in general, be non-immunogenic (Hewitt, Blake and Walder, 1976). However, WEHI-11, a spontaneous fibrosarcoma which arose in a BALB/c mouse, had TATA detectable *in vivo* and also expressed TAA *in vitro*. Furthermore, these tumour antigens were shared with 2 other carcinogen-induced fibrosarcomas.

As reviewed herein, cross-reacting tumour antigens have been detected on murine fibrosarcomas by both *in vivo* and *in vitro* techniques. When CL are induced *in vitro* to fibrosarcoma antigens it has been claimed that unique TAA dominate the response (McKhann and Jagarlamoody, 1971; Warnatz and Scheiffarth, 1974; Kall and Hellstrom, 1975; Kall *et al.*, 1976). However, in the studies presented herein, cross-reacting TAA were the major antigens detected, both *in vivo* and *in vitro*. Furthermore there is evidence that these techniques detect a heterogeneity of fibrosarcoma TAA. At least 2 cross-reacting TATA were detected *in vivo*, one expressed on both WEHI-11 and WEHI-164, and one expressed on WEHI-167 and WEHI-11. WEHI-167 grew equally well in mice immunized to any of the fibrosarcomas as in control mice; however, it did immunize mice to a shared TATA as detected by challenge with WEHI-11. This result illustrates a major paradox of tumour immunity, where despite the detection of TAA on many tumours a fatal outcome usually ensues for the host. A tumour may readily induce an immune response but be resistant to the effector immune mechanism. The ability of a tumour to immunize against shared TATA but grow just as rapidly in immune and non-immune mice has been reported for murine plasmacytomas (Burton and Warner, 1976). These *in vivo* results exemplify the need carefully to titrate tumours in the strain of origin, so that too large a challenge dose is not used.

The results of the *in vitro* induction of cytotoxic lymphocytes to these fibrosarcomas has demonstrated a complete sharing of antigens between the 3 tumours. The response as revealed by anti-Thy-1.2 serum treatment, is completely mediated by cytotoxic T lymphocytes, as was found for the *in vivo* response to the fibrosarcoma WEHI-164 (Rollinghoff and Warner, 1973). Since all 3 tumours completely inhibited the cytotoxic activity directed against one of them, a common antigenic determinant is involved. The degree of inhibition observed at blocker/target ratios of less than 30/1 is due to specific inhibition and not to the non-specific inhibition observed by these tumours at higher ratios (Fig. 6). The nature of this common antigenic determinant has not been fully defined, but is clearly shown to be distinct from the oncofoetal antigens also expressed by these tumours (Chism *et al.*, 1976) since foetal liver shows no significant blocking in this system, whereas it does so in the OFA studies (Chism *et al.*, 1976). The possibility that sensitization to foetal calf serum components is involved has been discussed elsewhere in detail (Burton *et al.*, 1977) but is not the explanation for this common antigen, since the WEHI-167 cells were derived from *in vivo* tumour, not from a cultured line.

This work was supported by the Wellcome Foundation UK, and is in part pursuant to Contract NO1-CB-23889 from the National Cancer Institute. R.C.B. is a NH and MRC postgraduate research scholar. Ms Dianne Grail and Mrs Judy Thompson provided excellent technical assistance.

### REFERENCES

BALDWIN, R. W. (1973) Immunological Aspects of Chemical Carcinogenesis. *Adv. Cancer Res.*, **18**, 1.

BATAILLON, G., ROSS, G. & KLEIN, G. (1975) Comparative *In vitro* Sensitivity of Two Methylcholanthrene-induced Murine Sarcoma Lines to Humoral and Cellular Immune Cytotoxicity. *Int. J. Cancer*, **16**, 255.

BURDICK, J. F. & WELLS, S. A. (1973) Cross-reactivity between Cell-surface Antigens of Different Murine Carcinogen induced Tumours, Demonstrated by a Modified Isotopic Antiglobulin Test. *J. natn. Cancer Inst.*, **51**, 1149.

BURTON, R., THOMPSON, J. & WARNER, N. L. (1975) *In vitro* Induction of Tumour-specific Immunity. I. Development of Optimal Conditions for Induction and Assay of Cytotoxic Lymphocytes. *J. Immun. Meth.*, **8**, 133.

BURTON, R. C. & WARNER, N. L. (1976) Tumor Immunity to Murine Plasma Cell Tumors. III. Detection of Common Tumor associated Antigens on BALB/c, C3H and NZB Plasmacytomas by *In vivo* and *In vitro* Induction of Tumor Immune Responses. *J. natn. Cancer Inst.*, **58**, 701.

BURTON, R. C., CHISM, S. E. & WARNER, N. L. (1976) *In vitro* Induction of Tumour specific Immunity. III. Lack of Requirements for H-2 Compatibility in Lysis of Tumor Targets by T Cells Activated *In vitro* to Oncofetal and Plasmacytoma Antigens. *J. Immun.*, **118**, 971.

BURTON, R. C., CHISM, S. E. & WARNER, N. L. (1977) *In vitro* Induction of Tumor specific Immunity VIII. Does Autosensitisation Occur with *In vitro* Culture of T Lymphocytes? *J. Immun.* (in press).

CHISM, S., BURTON, R. C. & WARNER, N. L. (1976) *In vitro* Induction of Tumor specific Immunity. II. Activation of Cytotoxic Lymphocytes to Murine Oncofetal Antigens. *J. natn. Cancer Inst.*, **57**, 377.

CHISM, S. E., BURTON, R. C., GRAIL, D., BELL, P. M. & WARNER, N. L. (1977) *In vitro* Induction of Tumour specific Immunity. VI. Analysis of Specificity of Immune Response by Cellular Competitive Inhibition: Limitations and Advantages of the Technique. *J. Immun. Meth.*, **16**, 245.

COHEN, I. R., GLOBERSON, A. & FELDMAN, M. (1971) Autosensitisation *In vitro*. *J. exp. Med.*, **133**, 834.

FOLEY, E. J. (1953) Antigenic Properties of Methylcholanthrene-induced Tumours in Mice of the Strain of Origin. *Cancer Res.*, **13**, 835.

FORBES, J. R., NAKAO, Y. & SMITH, R. T. (1975) Tumor-specific Immunity to Chemically Induced Tumors. Evidence of Immunologic Specificity and Shared Antigenicity in Lymphocyte Responses to Soluble Tumor Antigens. *J. exp. Med.*, **141**, 1181.

FRITZE, D., KERN, D. H., HUMME, J. A., DROGEMULLER, C. R. & PILCH, Y. H. (1976) Detection of Private and Common Tumor-associated Antigens in Murine Sarcomas Induced by Different Chemicals. *Int. J. Cancer*, **17**, 138.

GROSS, L. (1943) Intradermal Immunization of C3H Mice against a Sarcoma that Originated in an Animal of the Same Line. *Cancer Res.*, **3**, 326.

HARDER, F. H. & MCKHANN, C. F. (1968) Demonstration of Cellular Antigens on Sarcoma Cells by an Indirect $^{125}$I-labelled Antibody Technique. *J. natn. Cancer Inst.*, **40**, 231.

HELLSTRÖM, I., HELLSTRÖM, K. E. & PIERCE, C. (1968) *In vitro* Studies of Immune Reactions against Autochthonous and Syngeneic Mouse Tumours Induced by Methyl Cholanthrene and Plastic Discs. *Int. J. Cancer*, **3**, 467.

HEWITT, H. B., BLAKE, E. R. & WALDER, A. S. (1976) A Critique of the Evidence for Active Host Defence against Cancer, Based on Personal Studies of 27 Murine Tumours of Spontaneous Origin. *Br. J. Cancer*, **33**, 241.

ILFELD, D., CARNAUD, C., COHEN, I. R. & TRAININ, N. (1973) *In vitro* Cytotoxicity and *In vivo* Tumor Enhancement Induced by Mouse Spleen 213.

ILFELD, D., CARNAUD, C. & KLEIN, E. (1975) Cytotoxicity of Autosensitized Target Restricted to the H-2K End of Targets. *Immunogenetics*, **2**, 231.

KALL, M. A. & HELLSTRÖM, I. (1975) Specific Stimulatory and Cytotoxic Effects of Lymphocytes Sensitised *In vitro* to either Alloantigens or Tumour Antigens. *J. Immunol.*, **114**, 1083.

KALL, M. A., HELLSTRÖM, I. & HELLSTRÖM, K. E. (1976) *In vitro* Generation of Primary and Secondary Cytotoxic Cell mediated Immune Responses to Chemically Induced Mouse Sarcomas. *Int. J. Cancer*, **18**, 488.

KEARNEY, R., BASTEN, A. & NELSON, D. S. (1975) Cellular Basis for the Immune Response to Methylcholanthrene-induced Tumours in Mice. Heterogeneity of Effector Cells. *Int. J. Cancer*, **15**, 438.

KLEIN, G., SJØGREN, H. O., KLEIN, E. & HELLSTRÖM, K. E. (1960) Demonstration of Resistance against Cholanthrene induced Sarcomas in the Primary Autochthonous Host. *Cancer Res.*, **20**, 1561.

KOLDOVSKY, P. & SVOBODA, J. (1963) Cross-reaction between Benzypyrene-induced Tumours in Rats and Mice. *Folia Biol.* (*Prague*), **9**, 223.

McKhann, C. F. & Jagarlamoody, S. M. (1971) Evidence for Immune Reactivity against Neoplasms. *Transplant. Rev.*, **7**, 55.

Old, L. J., Boyse, E. A., Clarke, D. A. & Carsewell, E. A. (1962) Antigenic Properties of Chemically Induced Tumours. *Ann. N.Y. Acad. Sci.*, **101**, 80.

Prehn, R. T. & Main, J. M. (1957) Immunity to Methylcholanthrene-induced Sarcomas. *J. natn. Cancer Inst.*, **55**, 189.

Prehn, R. T. (1975) The Relationship of Tumor Immunogenicity to the Concentration of the Inducing Oncogen. *J. natn. Cancer Inst.*, **55**, 189.

Reiner, J. & Southam, C. M. (1967) Evidence of Common Antigenic Properties in Chemically Induced Sarcomas of Mice. *Cancer Res.*, **27**, 1243.

Robert, F., Oth, D. A. & Dumont, F. (1973) Cross-immunity between Chemically-induced Sarcomas, Detected by Transplantation in Restricted Genetic Conditions. *Eur. J. Cancer*, **9**, 877.

Rollinghoff, M. & Wagner, H. (1973) *In vitro* Induction of Tumor specific Immunity. Requirements for T Lymphocytes and Tumor Growth Inhibition *In vivo*. *Eur. J. Immun.*, **3**, 471.

Rollinghoff, M. (1974) Secondary Cytotoxic Tumor Response Induced *In vitro*. *J. Immun.*, **112**, 1718.

Rollinghoff, M. & Warner, N. L. (1973) Specificity of *In vivo* Tumour Rejection Assessed by Mixing Immune Spleen Cells with Target and Unrelated Tumour Cells. *Proc. Soc. exp. biol. Med.*, **144**, 813.

Rollinghoff, M., Rouse, B. T. & Warner, N. L. (1973) Tumour Immunity to Murine Plasma Cell Tumours. I. Tumour associated Transplantation Antigens of NZB and BALB/c Plasma Cell Tumours. *J. natn. Cancer Inst.*, **50**, 159.

Sanderson, C. J. (1976) The Mechanism of T Cell mediated Cytotoxicity II. Morphological Studies of Cell Death by Time Lapse Microcinematography. *Proc. R. Soc. Lond. B.*, **192**, 241.

Small, M. & Trainin, N. (1975) Inhibition of Syngeneic Fibrosarcoma Growth by Lymphocytes Sensitized on Tumour Cell Monolayers in the Presence of the Thymic Humoral factor. *Int. J. Cancer*, **15**, 962.

Tachibana, T. & Klein, E. (1970) Detection of Cell Surface Antigens on Monolayer Cells. I. The Application of Immune Adherence on a Microscale. *Immunology*, **19**, 711.

Takasugi, M. & Klein, E. (1970) A Microassay for Cell mediated Immunity. *Transplantation*, **9**, 219.

von Boehmer, H. & Shortman, K. (1973) The Separation of Different Cell Classes from Lymphoid Organs. IX. A Simple and Rapid Method for Removal of Damaged Cells from Lymphoid Cell Suspensions. *J. Immun. Meth.*, **2**, 293.

Wahl, D. V., Chapman, W. H., Hellström, I. & Hellström, K. E. (1974) Transplantation Immunity to Individually Unique Antigens of Chemically Induced Bladder Tumours in Mice. *Int. J. Cancer*, **14**, 114.

Warnatz, H. & Scheiffarth, F. (1974) Cell-mediated Immune Response of *In vitro* Sensitized Lymphocytes to Isogeneic Methyl-cholanthrene Induced Tumour Cell Lines. *Transplantation*, **18**, 273.

Whitney, R. B., Levy, J. G. & Smith, A. G. (1975) Studies on the Effector Cell of Anti-tumor Immunity in a Chemically Induced Mouse Tumour System. *Br. J. Cancer*, **31**, 157.

Winn, H. J. (1961) Immune Mechanisms in Homotransplantation. II. Quantitative Assay of the Immunologic Activity of Lymphoid Cells Stimulated by Tumour Homografts. *J. Immun.*, **86**, 228.